# Supplementary material for: Unilateral Lung Agenesis: A Systematic Review of Prevalence, Anatomical Variants, and Clinical Implications
Source: Diagnostics (Basel). 2025 Sep 8;15(17):2272. doi: 10.3390/diagnostics15172272 (PMC12428195; doi:10.3390/diagnostics15172272)
Supplement: Supplementary file 1 [file diagnostics-15-02272-s001.zip › diagnostics-3790614-supplementary.pdf]

Supplementary material

Table S1. Searches strategies

Database	Search strategy	Results
MEDLINE	"unilateral lung"[Title/Abstract] AND "Agenesia"[Title/Abstract]	1
MEDLINE	"unilateral lung"[Title/Abstract] AND "Aplasia"[Title/Abstract]	6
MEDLINE	"unilateral lung"[Title/Abstract] AND "Agenesis"[Title/Abstract]	40
SCOPUS	Unilateral lung absence	6029
WOS	“Unilateral lung” (Title) AND “Absence” (Title)	19
CINAHL	Unilateral lung” (Title) AND “Agenesia” (Title)	7
	Total	6102

* All searches were carried out on January 28th, 2024.

Table 2. Summary of the included articles.

**Author, year**	**Country; ethicity**	**Type of study**	**Total number of subjects**	**Age or Mean Age (SD) and / or range**	**Study technique**	**ULA's prevalence**	**Left ULA**	**Right ULA**	**Sex sample**
Ahmed et al., 2020	Saudi Arabia, not reported	Case report	1	24 years old	Chest X-ray, Thoracic CT scan	0	0	1	Female
Weber et al., 2023	USA, not reported	Case report	3	Neonates (weeks)	Chest X ray, CTA scan, flexible bronchoscopy	0	1	2	Male
Backer et al., 2009	USA, not reported	Case series	71	13 days; 3.5 years	Chest X-ray, axial CT, 3D CT reconstruction	11 silge lung ; 60 bilateral lung	0	0	Male and female
Ito et al., 2021	Japan, asian	Case report	1	Neonatal (37 weeks)	Transthoracic echocardiography and computer tomography	1	0	1	Female
Gorla et al., 2018	USA, not reported	Case report	1	35 weeks	Chesy X ray, CTA scan, echocardiogram, MRI	1	1	0	Female
Fitoz et al., 2001	Turkey, not reported	Case report	1	Neonates (weeks)	echocardiography, chest x-ray, CT	1	1	0	female
Nguyen et al., 2017	USA, not reported	Case report	1	14 years old	Chest CT with intravenous contrast,	3	0	1	Female

					cardiatic magnetic resonance imaging.				
Biswas et al., 2019	India, not reported	Case report	1	15 years old	Chest X-ray, Thoracic CT scan	3	0	1	Male
Tanrivermis Sayit & Elmi 2020	Turkey, not reported	Case report	1	53 years. old	Anteroposterior Chest X-ray, MDCT, echocardiography	1	0	1	Male
Sadiqi & Hamidi 2018	Afghanistan, not reported	Case report	6	3 month-18 years old	Contrast chest CT	Two patients-> Type I<br><br>Three patients -> Type II<br><br>One patient -> Type III	1	5	5 male and 1 female
Krivchenya et al., 2000	Ukraine, not reported	Case report	1	10 months-4 years old	Chest radiograph, cardiopulmonogram, tracheobronchoscopy	Type II	0	0	Female
Colson & Mortelliti 2005	USA, not reported	Case report	1	11 months	bronchoscopy angiography chest CT	3	0	1	male
Uttarwar et al., 2014	India, not reported	Case report	1	3 day-old	Chest X-Ray, bronchoscopy, angiography	1	0	1	Female
He et al., 2016	China, not reported	Case report	2	13-month-old/ 3-years-old	Electrocardiography, Transthoracic echocardiography, Thoracic	13 month = 1<br><br>3 years = 1	1	1	1 Female.<br><br>1 Male.

					CT, CT angiography, Eco Doppler				
Russell et al., 2014	USA, not reported	Case series	1	Minutes (case 3)	Echocardiogram	0	0	0	female
Labovsky et al., 2016	USA, not reported	Case report	1	38 4/7 weeks	Near-infrared spectroscopy, chest computed tomography	1	1	0	Female
Upadhyay H et al., 2016	USA, not reported	Case report	1	28 years old	Chest x-ray, Contrast-enhanced CT and echocardiogram.	1	0	1	Female
Thomsen, 1948	Denmark, not reported	Case report	1	36 years	tomograohie ;x-ray	1	1	0	Female
Kumar et al., 2016	India, not reported	Case report	1	40 years.old	Chest x- ray, Chest CT, CT pulmonary angiography, bronchoscopy, Two Dimensional Echocardiography (2D ECHO)	1	1	0	Female
Nandalike et al., 2011	USA, not reported	Case report	1	18-month-old	Chest radiograph, flexible bronchoscopy	2	0	1	Female
Kayemba-Kay's et al., 2013	France, not reported	Case report	4	Case 1: 37 weeks<br>Case 2: 37 weeks<br>Case 3: 32 weeks	Fetal MRI, fetal echocardiography and chest X-rays	1	1 (case 1)	3 (case 2, 3 and 4)	Case 1: Male<br>Case 2: Male<br>Case 3: Female

				Case 4: 39 weeks					Case 4: Male
Pierron C et al., 2011	USA, not reported	Case report	2	Subject 1: 4 months ; Subject 2: 2 years old	Chest X-ray, Dual source CT scanner, Echocardiography	Both patients had right lung aplasia (type II)		0	0	Subject 1 & 2: female
Gopinathan et al., 2011	United Kingdom, not reported	Case report	1	13 years old	bronchoscopy, echocardiogram, Chest x-ray	1	0	1	Female
Heffron et al., 2006	USA, not reported	Case report	1	Neonatal-3 and 1/2 months	Chest X-ray, echocardiogram, Magnet resonance imaging	2	0	1	Female
Bromley et al., 1997	USA, not reported	Case report	3	Case 1: Prenatal-32 weeks of gestation. Case 2: Prenatal-36 weeks of gestation. Case 3: Prenatal-19 weeks of gestation	Ultrasonography	Case 1: 2. Case 2: 2. Case 3: 1	3	0	--
Sicuranza & Figueroa, 2004	USA, not reported	Case report	1	33 years old	Chest X-ray, Echocardiogram, Echo-gated cardiac magnetic resonance imaging	1	0	1	Female

Kalache K.D et al., 1997	Germany, not reported	Case report	1	37 weeks of gestattion	Ultrasound examination, color doppler, chest x ray, cardiac echocardiography, thoracic CT	0	0	1	Male
Bentsianov et al., 2000	USA, Hispanic	Case report	2	1st subject: 5-years-old.<br>2nd subject: 4-week-old.	1st Subject: Chest radiograph, rigid brochoscopy , computed tomographic scan, echocardiogram and renal ultrasound.<br>2nd Subject: Chest radiograph, brochoscopy, computed tomographic scan with contrast. An echocardiogram and a renal ultrasound.	1st Subject: 1<br>2nd Subject:	(1)Subject 1	(1)Subject 2	1st Subject: Female<br>2nd Subject: Female
Downard et al., 2004	USA, not reported	Case report	1	35 weeks of gestation	Chest x-ray, abdominal ultrasound, echocardiogram, chest computed tomography	1	0	1	Female
Thomas et al., 1998	India, not reported	Case report	4	Subject 1: 1 year old Subject 2: 9 months Subject 3: 2 years old	Chest radiograph, thoracic CT, Bronchoscopy	Subject 1 and 2: type III; Suject 3: type II; Subject 4: type II	0	0	Subjects 1,2,3: male Subject 4: female

				Subject 4: 1.5 years old					
Abel, 1989	Germany, not reported	Case report	1	6 months	Thoracic CT, Bronchoscopy	Schneider type II (in this case right lung aplasia)	0	0	Female
Zhang Y et al., 2013	China, not reported	Case series	18	Mean age: 28 weeks (ages ranged form 18 to 35 weeks)	Postnatal color doppler echocardiography (CDE), digital radiography (DR), CT scann and autopsy findings	Schneider type I for all subjects	7 subjects	11 subjects	Not specified

Table 3. Risk of bias assessment according to the JBI critical appraisal check-list.

Author	JBI Q1	JBI Q2	JBI Q3	JBI Q4	JBI Q5	JBI Q6	JBI Q7	JBI Q8	Bias risk
Ahmed et. al., 2020	🟢	🟢	🟢	🟢	N/A	N/A	N/A	🔴	Moderate
Pierron et. al., 2011	🟢	🟢	🟢	🟢	🟢	🟢	N/A	🟢	Low
Weber et. al., 2023	🟢	🟢	🟢	🟢	🟢	🟢	🟢	🟢	Low
Backer et. al., 2009	🟢	🟢	🟢	🟢	🟢	🟢	🟢	🟢	Low
Ito et. al., 2021	🟢	🟢	🟢	🟢	🟢	🟢	🟢	🟢	Low
Gorla et. al., 2018	🔴	🟢	🟢	🟢	🟢	🟢	N/A	🟢	Low
Fitoz et. al., 2001	🔴	🟢	🟢	🟢	N/A	N/A	N/A	🟢	Low
Nguyen et al., 2017	🔴	🟢	🟢	🟢	🟢	🔴	🔴	🔴	Moderate
Uttarwar et. al., 2014	🔴	🟢	🟢	🟢	🟢	🟢	🟢	🟢	Low
Bromley et. al., 1997	🔴	🟢	🟢	🟢	🟢	🟢	🟢	🟢	Low
Labovsky et. al., 2016	🔴	🟢	🟢	🟢	🟢	🔴	🔴	🟢	Moderate
Kumar et. al., 2016	🔴	🟢	🟢	🟢	🟢	🟢	🟢	🔴	Low
Tanrivermis Sayit & Elmali. 2020	🔴	🟢	🟢	🟢	🟢	N/A	🔴	🟢	Moderate
Thomsen, 1948	🔴	🔴	🔴	🟢	🟢	🟢	🟢	🟢	Moderate
Sadiqi et Hamidi	🔴	🟢	🟢	🟢	N/A	N/A	N/A	🟢	Low
Biswas et. al., 2019	🔴	🟢	🟢	🟢	🟢	🟢	🟢	🔴	Low
Zhang Y. et. al., 2013	🔴	🟢	🟢	🟢	🟢	N/A	N/A	🟢	Moderate
He et. al., 2016	🔴	🟢	🟢	🟢	N/A	N/A	🟢	🟢	Low
Sicuranza & Figueroa, 2004	🟢	🟢	🟢	🟢	🟢	🟢	🟢	🟢	Low
Kayemba-Kay's et. al., 2013	🔴	🟢	🟢	🟢	🟢	🔴	🔴	🟢	Moderate
Hackell RS et. al., 2011	🟢	🟢	🟢	🟢	🟢	🟢	🟢	🟢	Low
Heffron et. al., 2006	🟢	🟢	🟢	🟢	🟢	🟢	🟢	🟢	Low

Colson & Mortelliti 2005									low
Krivchenya et. al., 2000									Low
Nandalike N. et. al., 2011									Moderate
Kalache K.D. et. al., 1997					N/A	N/A	N/A		Low
Hinesh U. et. al., 2016						N/A			Low
Bentsianov L. Boris et. al., 2000					N/A				Low
Thomas et. al., 1998									Low
Abel, 1989							N/A	N/A	Low
Russell. Et al 2014									High
Downard et. al., 2004									Low

Table S4: PRISMA Checklist

Section and Topic	Item #	Checklist item	Location where item is reported
**TITLE**			
Title	1	Identify the report as a systematic review.	Title page
**ABSTRACT**			
Abstract	2	See the PRISMA 2020 for Abstracts checklist.	Abstract
**INTRODUCTION**			
Rationale	3	Describe the rationale for the review in the context of existing knowledge.	1. Introduction
Objectives	4	Provide an explicit statement of the objective(s) or question(s) the review addresses.	1. Introduction
**METHODS**			
Eligibility criteria	5	Specify the inclusion and exclusion criteria for the review and how studies were grouped for the syntheses.	2.2. Elegibility criteria
Information sources	6	Specify all databases, registers, websites, organisations, reference lists and other sources searched or consulted to identify studies. Specify the date when each source was last searched or consulted.	2.3. Electronic search
Search strategy	7	Present the full search strategies for all databases, registers and websites, including any filters and limits used.	2.3. Electronic search
Selection process	8	Specify the methods used to decide whether a study met the inclusion criteria of the review, including how many reviewers screened each record and each report retrieved, whether they worked independently, and if applicable, details of automation tools used in the process.	2.4. Study selection
Data collection process	9	Specify the methods used to collect data from reports, including how many reviewers collected data from each report, whether they worked independently, any processes for obtaining or confirming data from study investigators, and if applicable, details of automation tools used in the process.	2.5. Data collection process
Data items	10a	List and define all outcomes for which data were sought. Specify whether all results that were compatible with each outcome domain in each study were sought (e.g. for all measures, time points, analyses), and if not, the methods used to decide which results to collect.	2.2. Elegibility criteria
	10b	List and define all other variables for which data were sought (e.g. participant and intervention characteristics, funding sources). Describe any assumptions made about any missing or unclear information.	2.2. Elegibility criteria
Study risk of bias assessment	11	Specify the methods used to assess risk of bias in the included studies, including details of the tool(s) used, how many reviewers assessed each study and whether they worked independently, and if applicable, details of automation tools used in the process.	2.6. Methodological Quality Assessment of the Included Studies
Effect measures	12	Specify for each outcome the effect measure(s) (e.g. risk ratio, mean difference) used in the synthesis or presentation of results.	N/A
Synthesis methods	13a	Describe the processes used to decide which studies were eligible for each synthesis (e.g. tabulating the study intervention characteristics and comparing against the planned groups for each	2.4. Study selection

Section and Topic	Item #	Checklist item	Location where item is reported
		synthesis (item #5)).	
	13b	Describe any methods required to prepare the data for presentation or synthesis, such as handling of missing summary statistics, or data conversions.	2.4. Study selection
	13c	Describe any methods used to tabulate or visually display results of individual studies and syntheses.	2.5. Data collection process
	13d	Describe any methods used to synthesize results and provide a rationale for the choice(s). If meta-analysis was performed, describe the model(s), method(s) to identify the presence and extent of statistical heterogeneity, and software package(s) used.	2.5. Data collection process
	13e	Describe any methods used to explore possible causes of heterogeneity among study results (e.g. subgroup analysis, meta-regression).	N/A
	13f	Describe any sensitivity analyses conducted to assess robustness of the synthesized results.	N/A
Reporting bias assessment	14	Describe any methods used to assess risk of bias due to missing results in a synthesis (arising from reporting biases).	2.5. Data collection process
Certainty assessment	15	Describe any methods used to assess certainty (or confidence) in the body of evidence for an outcome.	N/A
**RESULTS**			
Study selection	16a	Describe the results of the search and selection process, from the number of records identified in the search to the number of studies included in the review, ideally using a flow diagram.	3.1. Included Articles
	16b	Cite studies that might appear to meet the inclusion criteria, but which were excluded, and explain why they were excluded.	3.1. Included Articles
Study characteristics	17	Cite each included study and present its characteristics.	3.2. Characteristics of the Included Studies and the Study Population
Risk of bias in studies	18	Present assessments of risk of bias for each included study.	3.3. Methodological Quality Assessment of the Included Studies
Results of individual studies	19	For all outcomes, present, for each study: (a) summary statistics for each group (where appropriate) and (b) an effect estimate and its precision (e.g. confidence/credible interval), ideally using structured tables or plots.	N/A
Results of syntheses	20a	For each synthesis, briefly summarise the characteristics and risk of bias among contributing studies.	3.2. Characteristics of the Included Studies and the Study Population; and 3.3. Methodological Quality Assessment of the Included Studies
	20b	Present results of all statistical syntheses conducted. If meta-analysis was done, present for each the summary estimate and its precision (e.g. confidence/credible interval) and measures of statistical heterogeneity. If comparing groups, describe the direction of the effect.	N/A
	20c	Present results of all investigations of possible causes of heterogeneity among study results.	N/A
	20d	Present results of all sensitivity analyses conducted to assess the robustness of the synthesized results.	N/A
Reporting biases	21	Present assessments of risk of bias due to missing results (arising from reporting biases) for each synthesis assessed.	3.3. Methodological Quality Assessment of the Included Studies
Certainty of evidence	22	Present assessments of certainty (or confidence) in the body of evidence for each outcome assessed.	N/A
**DISCUSSION**			
Discussion	23a	Provide a general interpretation of the results in the context of other evidence.	Discussion
	23b	Discuss any limitations of the evidence included in the review.	Discussion
	23c	Discuss any limitations of the review processes used.	Discussion
	23d	Discuss implications of the results for practice, policy, and future research.	Discussion
**OTHER INFORMATION**			
Registration and protocol	24a	Provide registration information for the review, including register name and registration number, or state that the review was not registered.	2.1. Protocol
	24b	Indicate where the review protocol can be accessed, or state that a protocol was not prepared.	Protocol wasn't published
	24c	Describe and explain any amendments to information provided at registration or in the protocol.	N/A
Support	25	Describe sources of financial or non-financial support for the review, and the role of the funders or sponsors in the review.	Funding
Competing interests	26	Declare any competing interests of review authors.	Competing interest
Availability of data, code and other materials	27	Report which of the following are publicly available and where they can be found: template data collection forms; data extracted from included studies; data used for all analyses; analytic code; any other materials used in the review.	2.3. Electronic search
